# Oxotremorine treatment reduces repetitive behaviors in BTBR T+ tf/J mice

**DOI:** 10.3389/fnsyn.2014.00017

**Published:** 2014-08-13

**Authors:** Dionisio A. Amodeo, Julia Yi, John A. Sweeney, Michael E. Ragozzino

**Affiliations:** ^1^Laboratory of Integrative Neuroscience, Department of Psychology, University of Illinois at ChicagoChicago, IL, USA; ^2^Department of Psychiatry, University of Texas Southwestern Medical CenterDallas, TX, USA

**Keywords:** acetylcholine, muscarinic receptors, autism, repetitive behaviors, marble burying, grooming

## Abstract

Repetitive behaviors with restricted interests is one of the core criteria for the diagnosis of autism spectrum disorder (ASD). Current pharmacotherapies that target the dopaminergic or serotonergic systems have limited effectiveness in treating repetitive behaviors. Previous research has demonstrated that administration of muscarinic cholinergic receptor (mAChR) antagonists can exacerbate motor stereotypies while mAChR agonists reduce stereotypies. The present study determined whether the mAChR agonist, oxotremorine affected repetitive behaviors in the BTBR T+ tf/J (BTBR) mouse model of autism. To test the effects of oxotremorine on repetitive behaviors, marble burying and grooming behavior were measured in BTBR mice and compared to that in C57BL/6J (B6) mice. The effects of oxotremorine on locomotor activity was also measured. Thirty minutes before each test, mice received an intraperitoneal (ip) injection of saline, 0.001 mg or 0.01 mg of oxotremorine methiodide. Saline- treated BTBR mice exhibited increased marble burying and self-grooming behavior compared to that of saline-treated B6 mice. Oxotremorine significantly reduced marble burying and self-grooming behavior in BTBR mice, but had no significant effect in B6 mice. In addition, oxotremorine did not affect locomotor activity in BTBR mice, but significantly reduced locomotor activity in B6 mice at the 0.01 mg dose. These findings demonstrate that activation of mAChRs reduces repetitive behavior in the BTBR mouse and suggest that treatment with a mAChR agonist may be effective in reducing repetitive behaviors in ASD.

## Introduction

Autism spectrum disorders (ASDs) represent a cluster of neurodevelopmental disorders characterized by social and communicative impairments, as well restricted interests and repetitive behaviors (RRBs). RRBs are subdivided into lower-order and high-order behaviors (Lam and Aman, 2007). Lower order RRBs involve repetitive manipulation of objects, stereotyped movements or repetitive self-injurious behavior (Lam and Aman, 2007). Higher order RRBs are characterized by an insistence on sameness, or rigid adherence to a rule or routine (Turner, 1999; Szatmari et al., 2006). RRBs are reported to be the most distressing aspect of ASD for patients and families that profoundly impact daily living (Bishop et al., 2007).

Current treatments for RRBs have limited effectiveness (Boyd et al., 2012). Most pharmacotherapies in ASD focus on treating symptoms by principally modifying dopaminergic and serotonergic signaling (McPheeters et al., 2011). Atypical antipsychotics have food and drug administration (FDA) indications for treating irritability but not the core features of ASD (McPheeters et al., 2011). Selective serotonin reuptake inhibitor (SSRI) medications, used to reduce restricted interest symptoms, have resulted in mixed improvements with irritability observed in some individuals (Hollander et al., 2005; Owley et al., 2005, 2010; Henry et al., 2006; King et al., 2009). An alternative possibility is to treat RRBs by targeting the cholinergic system. Accumulating evidence suggests that brain cholinergic abnormalities could explain some of the pathophysiology in ASD. Post-mortem studies indicate that there is altered expression of muscarinic and nicotinic cholinergic receptors (Perry et al., 2001; Deutsch et al., 2010). Recent gene networks that confer risk for ASD include genes related to cholinergic transmission and these are also highly expressed in the brain (Voineagu et al., 2011; Ben-David and Shifman, 2012; Lee et al., 2012). Moreover, anti-psychotic treatments which have significant muscarinic receptor antagonism, e.g., quetiapine, can exacerbate symptoms in ASD (Martin et al., 1999; Hardan et al., 2005). Thus, treatments that increase muscarinic cholinergic receptor (mAChR) transmission may reduce core symptoms in ASD.

Animal models are often an important initial step in evaluating new treatment approaches. The BTBR T+ tf/J (BTBR) mouse is one preclinical model employed to better understand ASD because the mouse exhibits a phenotype that is comparable to the core symptoms in ASD (see Meyza et al., 2013 for review). Compared to B6 mice, BTBR mice exhibit deficits in social interactions and communication (McFarlane et al., 2008; Scattoni et al., 2008, 2011; Pobbe et al., 2010; Silverman et al., 2010; Chadman, 2011); restricted interests and behavioral inflexibility (Moy et al., 2008; Pearson et al., 2011; Amodeo et al., 2012; Karvat and Kimchi, 2012; Guariglia and Chadman, 2013); as well as repetitive or stereotyped behaviors, e.g., increased self-grooming and marble burying (McFarlane et al., 2008; Silverman et al., 2010; Amodeo et al., 2012; Babineau et al., 2013; McTighe et al., 2013; Reynolds et al., 2013). Grooming and digging can be viewed as part of the common behavioral repertoire exhibited by rodents (Garner and Mason, 2002). Studying these behaviors in BTBR mice is of particular interest related to ASD because of the excessive quantity in which these behaviors are expressed (Yang et al., 2007, 2009; McFarlane et al., 2008; Pobbe et al., 2010; Pearson et al., 2011; Amodeo et al., 2012), as well as being exhibited in various contexts and with repeated testing (Yang et al., 2007, 2009; McFarlane et al., 2008; Pobbe et al., 2010). For example, Yang et al. (2007) found that BTBR mice showed higher levels of repetitive grooming than B6 mice when raised with either a biological BTBR mother, a foster BTBR mother, or a low grooming B6 mother, demonstrating that excessive self-grooming in BTBR mice is not attenuated by an environmental influence such as caregiver.

In addition, recent findings indicate that BTBR mice exhibit decreased brain acetylcholine levels (McTighe et al., 2013) and infusion of an acetylcholinestase inhibitor into the dorsomedial striatum can alleviate a reversal learning deficit in BTBR mice (Karvat and Kimchi, 2014). However, unknown is whether cholinergic treatments may also be effective in reducing repetitive motor behaviors in BTBR mice. A past study reported that treatment with a mAChR agonist can reduce stereotyped behaviors in rodents (Wang and McGinty, 1997). Moreover, post-mortem studies report reduced mAChR signaling in various brain regions of ASD patients (Deutsch et al., 2010). Thus, treatment with a mAChR agonist may be effective in reducing repetitive behaviors in ASD.

To determine whether treatment with a mAChR agonist reduces repetitive behaviors, the present experiments examined whether the mAChR agonist, oxotremorine methoidine alleviates elevated self-grooming and marble burying in BTBR mice compared to that in B6 mice. To understand whether oxotremorine treatment has a more general effect on motor behavior, the effect of oxotremorine on horizontal locomotor activity was also measured in BTBR and B6 mice.

## Materials and methods

### Animals

Male C57BL/6J and BTBR mice, 7–8 weeks old, were obtained from the Jackson Laboratory (Bar Harbor, ME). Mice were singly housed in plastic cages (28 cm wide × 17 cm long × 12 cm high) in humidity (30%) and temperature (22°C) controlled room with a 12-h light/dark cycle (lights on at 07:00 am). Ten to fourteen days after arrival behavioral testing procedures began. Animal care and use was in accordance with the National Institutes of Health Guide for the Care and Use of Laboratory Animals, and was approved by the Institutional Laboratory Animal Care and Use Committee at the University of Illinois at Chicago.

### Drugs

Oxotremorine methoidine (Tocris, Ellisville, MO) 0.001 and 0.01 mg/kg was dissolved in 0.9% physiological saline. Mice received an intraperitoneal (ip) injection at 10 ml/kg volume.

### Spontaneous self-grooming

The procedure used to measure spontaneous self-grooming behavior was modified from McFarlane et al. (2008). Mice were individually placed in a clear plastic cage (28 cm wide × 17 cm long × 12 cm high) for a total of 20 min. Twenty minutes before being placed in the plastic cage mice received an ip injection of either vehicle, 0.001 or 0.01 mg/kg of oxotremorine. The treatment groups included the following: B6-vehicle (*n* = 8), B6-0.001oxotremorine (*n* = 8), B6-0.01oxotremorine (*n* = 8), BTBR-vehicle (*n* = 9), BTBR-0.001oxotremorine (*n* = 9), BTBR-0.01oxotremorine (*n* = 9). These doses were chosen based on past studies measuring the effects of oxotremorine on activity in rodents (Yano et al., 2009; Koda et al., 2011). The plastic cage was placed in a room separate from the mouse housing room. Subjects were allowed to freely explore the cage for the entirety of the test. The first 10 min served as a habituation period. Therefore mice received injections 30 min prior to measurement of grooming behavior. During the second 10 min of testing a trained observer sat approximately 1.6 m from the test cage and recorded cumulative time spent grooming all body regions in real time with a stopwatch. Grooming behavior included head washing, body grooming, genital/tail grooming and paw and leg licking. Experimenters were blind to treatment but were not blind to strain because BTBR mice are dark brown with a cream colored ventral patch while B6 do not have this patch. After each mouse was tested, the cage was thoroughly cleaned with a 2% ammonium chloride solution.

### Marble burying

Subjects tested for grooming behavior were also tested for marble burying. The marble burying test occurred 8 days following the grooming test to ensure there were no potential residual effects from the initial drug treatment (Birdsall et al., 1978). Subjects received a different treatment before marble burying from that administered during the spontaneous self-grooming experiment, with the exception of three B6 mice. One B6 received vehicle in both tests and two B6 mice received oxotremorine 0.01 mg in both tests. For all other mice, approximately half from each treatment group in the self-grooming test were assigned to one of the other two treatment groups. For example, for mice receiving 0.001 mg oxotremorine in the self-grooming test, approximately half were assigned to the vehicle group and half assigned to the 0.01 mg oxotremorine group. With this experimental design, half of the mice in the oxotremorine treatment groups (low or high dose) were receiving the drug for the first time. Seven days after the grooming test, mice were habituated to the plastic container used for the marble burying test. The same marble burying test procedure was used as in Amodeo et al. (2012). Mice were individually placed in a plastic container (46 cm long by 24 cm wide by 21 cm deep) with 3 cm of clean woodchip bedding (Northeastern Products, NY). The plastic container was placed in a room used for behavioral testing. Mice were allowed to freely explore a container for 30 min undisturbed. This served to habituate mice to the chamber. Twenty-four hours later, 20 glass marbles (1.5 cm in diameter) were arranged in five rows of four. The marbles were placed on top of 3 cm of clean woodchip bedding. A template was used to ensure that there was a consistent positioning of marbles. Thirty minutes before being placed into the test container, mice received an injection of either vehicle, 0.001, or 0.01 mg of oxotremorine in 0.9% physiological saline. The treatment groups included the following: B6-vehicle (*n* = 8), B6-0.001oxo (*n* = 8), B6-0.01oxo (*n* = 8), BTBR-vehicle (*n* = 9), BTBR-0.001oxo (*n* = 9), BTBR-0.01oxo (*n* = 9). As in the grooming test, experimenters were blind to treatment but were not blind to strain. Once a mouse was placed into the test container a wire lid was placed on top. Mice were allowed to explore the container and marbles for 30 min. After 30 min, each mouse was removed from the testing container and returned to their home cage. Marbles were considered buried if ≥2/3 of the surface area was covered in woodchip bedding. The total number of buried marbles was recorded. Between testing, marbles were thoroughly cleaned and new bedding was used for each mouse.

### Locomotor activity

A separate group of naïve mice were used to measure locomotor activity. Testing of locomotor activity was conducted in a black acrylic rectangular-shaped chamber (76 cm long × 50 cm wide × 30 cm high). Mice were injected with vehicle, 0.001 or 0.01 mg of oxotremorine 30 min before being placed in the test chamber. Before mice were introduced to the testing chamber, the entire apparatus was cleaned with 2% ammonium chloride solution. Treatment groups included the following: B6-vehicle (*n* = 8), B6-0.001oxo (*n* = 8), B6-0.01oxo (*n* = 8), BTBR-vehicle (*n* = 8), BTBR-0.001oxo (*n* = 8), BTBR-0.01oxo (*n* = 8). The bottom of the chamber was divided into nine (25 × 16.5 cm) equally sized rectangles. After a mouse was placed into the chamber the experimenter exited the testing room for 20 min. Once the session ended mice were removed from the test chamber and returned to the vivarium. Locomotor activity was recorded via camcorder (Sony Handycam, model DCR-DVD650) stationed above the chamber. Once the testing session was complete, locomotor activity was measured by an observer blind to treatment conditions. The number of lines crossed was calculated. A line cross was defined as a mouse having all four paws cross a line. The number of lines crossed was calculated in two separate 10-min blocks.

### Statistical analysis

Separate two-way analysis of variance ANOVAs (*strain*: B6, BTBR × *treatment*: vehicle, 0.001, 0.01 mg/kg oxotremorine) were conducted for self-grooming and marble burying. A significant interaction was followed by Tukey HSD *post hoc* tests to determine significant treatment differences in both strains. A three-way ANOVA with repeated measures (*strain* × *treatment* × *block*) was conducted for locomotor activity. A significant interaction was followed by Tukey HSD *post hoc* tests.

## Results

### Spontaneous self-grooming

**Figure 1:**
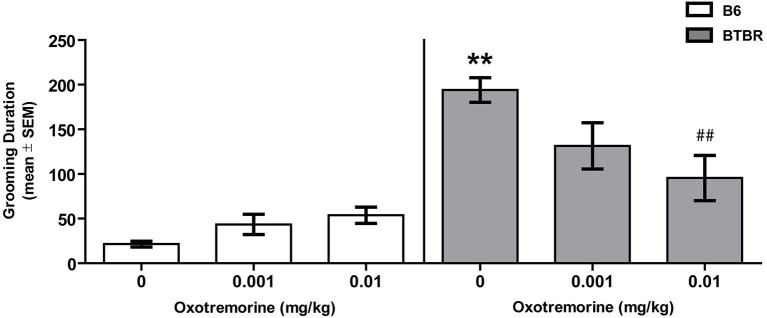
**Oxotremorine treatment attenuates spontaneous self-grooming behavior in BTBR mice**. Mean (±SEM) time spent grooming all body regions. Self-grooming behavior was measured in BTBR and B6 mice. Each mouse received an i.p. injection of vehicle, 0.001 or 0.01 mg of oxotremorine 30 min before grooming behavior was measured. BTBR mice spent significantly more time grooming compared to that of B6 mice. Vehicle or oxotremorine treatment did not affect spontaneous grooming behavior in B6 mice. Oxotremorine at 0.01 mg significantly decreased spontaneous grooming in BTBR mice. B6: vehicle (*n* = 8), 0.001 (*n* = 8), 0.01 (*n* = 8), BTBR: vehicle (*n* = 9), 0.001 oxo (*n* = 9), 0.01 oxo (*n* = 9). ** *p* < 0.01 vs. B6-vehicle, ## *p* < 0.01 vs. BTBR-vehicle.

Figure 1 illustrates the findings for spontaneous self-grooming in BTBR and B6 mice. Vehicle-treated BTBR mice spent approximately 180 s grooming compared to 20 s in B6 mice. Oxotremorine decreased self-grooming behavior in BTBR mice with the largest effect at the 0.01 mg dose which reduced the time self-grooming to half that observed in vehicle-treated BTBR mice. In contrast, oxotremorine treatment tended to increase self-grooming behavior in B6 mice. The main effect of strain was significant (*F*_(1,45)_ = 48.26, *p* < 0.01), but there was no significant treatment effect (*F*_(2,45)_ = 1.78, *p* > 0.05). However there was a significant strain × treatment interaction (*F*_(2,45)_ = 6.99, *p* < 0.01). *Post hoc* tests indicated that in the vehicle-treated groups, BTBR mice spent significantly more time self-grooming than that of B6 mice (*p* < 0.01). Oxotremorine 0.001 treatment in BTBR mice reduced self-grooming time, but the difference was not significantly different from that of vehicle-treated BTBR mice (*p* > 0.05). In contrast, oxotremorine 0.01 treatment significantly reduced self-grooming time compared to that of vehicle treatment in BTBR mice (*p* < 0.01). In B6 mice, oxotremorine treatment tended to increase in self-grooming time, but self-grooming time for both doses compared to that of vehicle treatment was not significant (*p*’s > 0.05). Thus, oxotremorine treatment reduced self-grooming behavior in BTBR mice in a dose-dependent fashion.

### Marble burying

**Figure 2:**
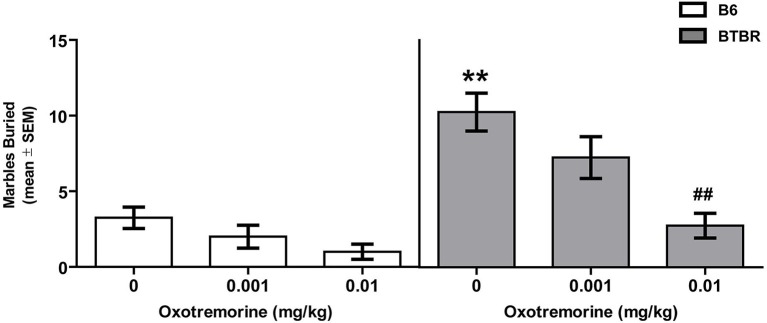
**Oxotremorine treatment attenuates marble burying in BTBR mice**. Mean (±SEM) marbles buried. Marble burying was measured in BTBR and B6 mice. Each mouse received an i.p. injection of vehicle, 0.001 or 0.01 mg of oxotremorine 30 min prior to marble exposure. BTBR mice buried significantly more marbles compared to that of B6 mice. Vehicle or oxotremorine treatment did not significantly affect marble burying in B6 mice. Oxotremorine at 0.01 mg significantly decreased marble burying in BTBR mice. B6: vehicle (*n* = 9), 0.001 oxo (*n* = 8), 0.01 oxo (*n* = 8), BTBR: vehicle (*n* = 9), 0.001 oxo (*n* = 9), 0.01 oxo (*n* = 9). ** *p* < 0.01 vs. B6-vehicle, ## *p* < 0.01 vs. BTBR vehicle.

The effects of oxotremorine treatment on marble burying behavior in BTBR and B6 mice are shown in Figure 2. Vehicle-treated BTBR buried approximately 10 marbles compared to 3 in B6 mice. Oxotremorine dose-dependently decreased marbles buried in BTBR mice. The oxotremorine 0.001 mg dose reduced marbles buried to approximately seven. The higher dose of oxotremorine reduce marble burying to approximately three. There was a significant main effect for strain (*F*_(1,42)_ = 35.87, *p* < 0.01) and treatment (*F*_(2,42)_ = 13.12, *p* < 0.01). Similarly, there was a significant strain × treatment interaction, (*F*_(2,42)_ = 3.92, *p* < 0.05). *Post hoc* tests indicated that in the vehicle-treated groups, BTBR mice buried significantly more marbles than that of B6 mice (*p* < 0.01). In BTBR mice, oxotremorine 0.001 mg treatment reduced marble burying, but the difference was not significantly different from that of vehicle treatment (*p* > 0.05). In contrast, oxotremorine 0.01 mg treatment significantly reduced marble burying compared to that of vehicle treatment in BTBR mice (*p* < 0.01). In B6 mice, there was a trend for oxotremorine treatment to reduce marble burying, although neither dose compared to that of vehicle treatment was significant (*p*’s > 0.05). Thus, oxotremorine treatment reduced marble burying in BTBR mice in a dose-dependent manner.

One possibility is that the prior treatment received in the self-grooming test interacted with the treatment received in the marble burying test to affect performance. As each treatment group in marble burying included mice that received a mixture of treatments this could be examined within each treatment group. In both the low-dose and high-dose oxotremorine group, BTBR mice that previously received vehicle treatment compared to mice that previously received the drug exhibited comparable marble burying performance. Specifically, in the oxotremorine 0.001 mg group, mice that previously received vehicle had a mean marble burying score of 6.75 ± 2.7 SEM while mice that previously received the drug had a mean score of 7.75 ± 1.3. In the oxotremorine 0.01 mg group, previous vehicle treatment led to a mean score of 3.0 ± 1.5 while previous drug treatment led to a mean score of 2.5 ± 0.9. In the vehicle-treated group, mice that previously received the low dose of oxotremorine had a mean score of 11.0 ± 1.3 while mice that previously received the high dose of oxotremorine had a mean score of 10.75 ± 0.6. Thus, previous treatment in the self-grooming test did not affect performance in the vehicle-treated group or drug groups during the marble burying test.

A similar pattern was observed for B6 mice. In the oxotremorine 0.001 mg group, mice that previously received vehicle had a mean marble burying score of 2.33 ± 0.58 SEM while mice that previously received the drug had a mean score of 2.25 ± 1.44. In the oxotremorine 0.01 mg group, previous vehicle treatment led to a mean score of 1.0 ± 0.41 while previous drug treatment led to a mean score of 1.33 ± 1.15. In the vehicle-treated group, mice that previously received the low dose of oxotremorine had a mean score of 3.25 ± 0.53 while mice that previously received the high dose of oxotremorine had a mean score of 3.67 ± 0.38.

### Locomotor activity

**Figure 3:**
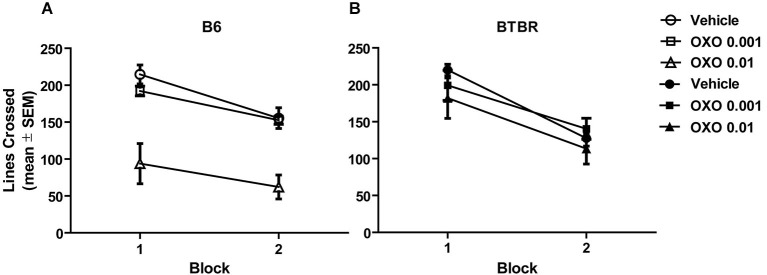
**Oxotremorine treatment did not affect locomotor activity in BTBR mice across two consecutive 10-min blocks**. Mean (±SEM) lines crossed. Locomotor activity was measured in B6 and BTBR mice. Each mouse received an i.p. injection of vehicle, 0.001 or 0.01 mg of oxotremorine 30 min before locomotor activity was measured. **(A)** Locomotor activity in B6 mice. Activity decreased in the second 10-min block compared to the first. Oxotremorine 0.01 mg significantly reduced locomotor activity compared to that of other treatment groups. B6: vehicle (*n* = 8), 0.001 oxo (*n* = 8), 0.01 oxo (*n* = 8). **(B)** Locomotor activity in BTBR mice. Activity decreased in the second 10-min block compared to the first. Oxotremorine treatment did not affect activity compared to that of vehicle treatment. BTBR: vehicle (*n* = 8), 0.001 oxo (*n* = 8), 0.01 oxo (*n* = 8).

Figures 3A,B illustrates the findings for locomotor activity in B6 and BTBR mice, respectively. The locomotor activity was analyzed across two 10 min blocks. All groups exhibited similar locomotor activity, with the exception of oxotremorine 0.01 mg in B6 mice. There was a significant effect of treatment (*F*_(2,42)_ = 18.82, *p* < 0.001), but there was no significant strain effect (*F*_(1,42)_ = 2.65, *p* > 0.05). However, the strain × treatment interaction was significant (*F*_(2,42)_ = 8.31, *p* < 0.01). *Post hoc* analysis revealed that in B6 mice, the oxotremorine 0.01 mg treatment significantly lowered activity compared to that of all other treatment groups (*p*’s < 0.05). The analysis further revealed that there was a significant effect for block (*F*_(1,40)_ = 110.01, *p* < 0.001), reflecting that mice decreased their activity in the second block compared to the first block. There was also a significant block × strain interaction (*F*_(1,40)_ = 5.88, *p* < 0.05). *Post hoc* tests revealed that block 2 activity in B6 mice was significantly reduced compared to block 1 activity in B6 and BTBR mice (*p*’s < 0.05). In addition, block 2 activity was significantly reduced compared to block 1 activity in BTBR mice (*p* < 0.05). No other interactions were significant.

## Discussion

Individuals with ASD exhibit repetitive, stereotyped behaviors and cognitive inflexibility that can severely limit daily living (Bishop et al., 2007; Lam and Aman, 2007; D’Cruz et al., 2013). Comparable to that observed in ASD, BTBR mice exhibited increased repetitive behaviors compared to that of B6 mice. The increased repetitive behaviors in BTBR mice included both elevated self-grooming and marble burying as observed in past studies (Yang et al., 2007; Silverman et al., 2010; Gould et al., 2011; Pearson et al., 2011; Amodeo et al., 2012). Because past studies in rats indicated that mAChR antagonists increase stereotyped behavior while mAChR agonists reduce stereotyped behavior (Wang and McGinty, 1997; Laviolette et al., 2000; Aliane et al., 2011), these studies investigated whether treatment with the non-specific, mAChR agonist, oxotremorine reduced repetitive behaviors in BTBR mice. Acute oxotremorine treatment, dose-dependently, attenuated the elevated self-grooming and marble-burying behavior in BTBR mice. These findings suggest that activation of mAChR can attenuate certain repetitive behaviors.

A past study examined the self-grooming microstructure in BTBR mice (Pearson et al., 2011). This analysis showed that BTBR mice exhibit an increase in almost all grooming subtypes with the exception of paw licking. Examination of the self-grooming microstructure also revealed that BTBR mice display a decrease in the percentage of incorrect transitions across the different grooming subtypes. Mice commonly groom in a cephalocaudal fashion starting with head washing and concluding with tail/genital licking. The present study did not examine the self-grooming microstructure or the grooming sequence. Thus, unknown is whether oxotremorine preferentially affected grooming subtypes or broadly decreased grooming subtypes. Further, unclear from the present study is whether oxotremorine altered the grooming sequence in BTBR mice in any way. Future studies investigating the effects of mAChR treatment on repetitive behaviors can address how mAChR agonists may alter self-grooming subtypes and self-grooming sequence. However, the present results indicate that oxotremorine treatment decreases self-grooming duration in BTBR mice without the highest dose of oxotremorine having an effect on locomotor activity. Taken together, the results suggest that mAChR agonist treatment may be effective in reducing lower-order repetitive behaviors in ASD.

In contrast to BTBR mice, B6 mice exhibited minimal grooming behavior as reported previously (Yang et al., 2007; McFarlane et al., 2008; Silverman et al., 2010; Pearson et al., 2011; Amodeo et al., 2012). Oxotremorine treatment actually showed a trend toward increasing grooming behavior in B6 mice. This increase in grooming behavior may explain why an oxotremorine injection in B6 mice tended to decrease marble burying and locomotor activity. The opposite effects of oxotremorine on grooming behavior in BTBR and B6 mice may suggest that there is an inverted U-shaped curve for mAChR activation to minimize grooming behavior. In particular, B6 mice may typically exhibit the “optimal level” of mAChR activity, but when treated with a mAChR agonist, i.e., oxotremorine, this increases mAChR activation above the optimal levels leading to increased grooming. Conversely, BTBR mice may have lower levels of mAChR activation leading to increased grooming, but treatment with oxotremorine brings mAChR activity into the optimal range that then decreases grooming behavior. Therefore, either too little or too great mAChR activation may lead to increased grooming behavior.

Comparable to that observed with self-grooming, oxotremorine 0.01 mg significantly reduced marble burying in BTBR mice. In B6 mice, there was a trend for oxotremorine 0.01 mg to reduce marble burying. Relative to BTBR mice, B6 mice display low levels of marble burying. The lower level of marble burying in B6 mice is consistent with previous studies (Amodeo et al., 2012; Schwartzer et al., 2013). However, because B6 mice exhibit a low level of marble burying this may obscure a drug effect in reducing marble burying. Another potential issue in interpreting the marble burying results is that mice were tested on marble burying following a self-grooming test. One possibility is that a previous treatment in the self-grooming test affected marble burying behavior. However, examination of the previous treatment received indicated that this did not influence marble burying behavior. This was the case for both BTBR mice and B6 mice. Also worth noting is that the number of marbles buried by vehicle-treated BTBR mice was comparable to that buried by drug-naïve BTBR mice in previous studies (Gould et al., 2011, 2012; Amodeo et al., 2012; Schwartzer et al., 2013). Thus, despite vehicle-treated BTBR mice in the marble burying test receiving either the low or high dose of oxotremorine in the self-grooming test, this did not alter their marble burying behavior compared to past observations in BTBR mice. Again, because oxotremorine treatment had no effect on locomotor activity in BTBR mice, the drug-induced reduction in marble burying behavior can not be explained by a more general reduction in activity. Instead, the results suggest that activation of mAChRs selectively modulated repetitive behaviors in BTBR mice.

The current findings complements a recent study that demonstrated treatment with the acetylcholinesterase inhibitor, donepezil, can improve behavioral rigidity as measured by reversal learning in BTBR mice (Karvat and Kimchi, 2014). Because acetylcholinesterase inhibitors leads to a non-specific increase in acetylcholine levels unknown is whether specific cholinergic receptors mediate these behavioral effects. The current experiments investigating the effects of oxotremorine demonstrate that activation of mAChRs is sufficient to attenuate repetitive behaviors in BTBR mice. Although stimulation of mAChRs was able to attenuate repetitive behaviors, this does not rule out that nicotinic cholinergic receptors may also play a role in affecting repetitive behaviors. Nicotine treatment in rats has shown to reduce certain stereotyped or repetitive behaviors (Zarrindast et al., 1999; Tizabi et al., 2002). Moreover, oxotremorine is a non-specific mAChR agonist, therefore still to be determined is whether specific mAChR subtypes may be sufficient to alleviate repetitive behaviors.

Previous studies investigated the effects of a M1 mAChR agonist on drug-induced or spontaneous grooming behavior in rodents (Bhattacharya and Sen, 1991; Inan et al., 2011). In both studies, McN-A-343 significantly reduced grooming behavior. Unclear is whether other muscarinic receptor subtypes may also be sufficient to reduce repetitive behaviors. There is evidence that targeting M5 mAChR can affect locomotion (Wang et al., 2004; Steidl and Yeomans, 2009), but unknown is whether this is restricted to general ambulation or also to motor stereotyped behavior. Repetitive behaviors in ASD have been separated into lower-order and higher-order repetitive behaviors (Bodfish et al., 2000; Lam and Aman, 2007). Lower order repetitive behaviors can include stereotyped movements or repetitive self-injurious behavior. Higher order RRBs instead are characterized by an “insistence on sameness” or rigid adherence to a rule or routine (Lam and Aman, 2007; Boyd et al., 2012). The findings with McN-343 suggest that treatment with a M1 mAChR agonist may be effective in treating lower-order repetitive behaviors in ASD. However, a recent study reported that the partial M1 mAChR agonist, CDD-102A, enhances set-shifting in rats (Ragozzino et al., 2012). Thus, treatment with a M1 mAChR agonist may be effective in treating both lower-order and higher-order repetitive behaviors.

The present studies indicated that a systemic injection of oxotremorine reduced repetitive behaviors in BTBR mice. The dorsomedial striatum may be a key anatomical site in which oxotremorine acts to affect repetitive behaviors. This is because drug treatments that increase stereotyped behaviors decrease acetylcholine output from this region (Aliane et al., 2011). Furthermore, destruction of cholinergic interneurons or injection of a mAChR antagonist in the dorsomedial striatum leads to increased repetitive behaviors that is alleviated by drug treatments that increase dorsomedial striatal acetylcholine output (Aliane et al., 2011). Cholinergic signaling in the dorsomedial striatum may not only be important for minimizing repetitive motor behaviors, but also for enabling cognitive flexibility. Karvat and Kimchi (2014) showed that donepezil injections into the dorsomedial striatum also improved reversal learning in BTBR mice. This effect of donepezil is consistent with past results showing that enhancing acetylcholine efflux in the rat dorsomedial striatum improves reversal learning while blocking acetylcholine efflux in this region impairs reversal learning (Palencia and Ragozzino, 2006; Ragozzino et al., 2009). Moreover, recent findings suggest that activation of M1 mAChRs in the dorsomedial striatum may mediate acetylcholine effects on cognitive flexibility (Tzavos et al., 2004; McCool et al., 2008; Ragozzino et al., 2012). Thus, treatment with a mAChR agonist may be effective in alleviating stereotyped motor behaviors and cognitive flexibility deficits.

To date, there exists some evidence, but not extensive findings, suggesting altered brain cholinergic transmission in ASD. In particular, there are results from gene networks that confer risk of ASD that include genes related to cholinergic transmission (Voineagu et al., 2011; Ben-David and Shifman, 2012; Lee et al., 2012) and post-mortem studies indicating reduced brain mAChR expression in ASD individuals (Deutsch et al., 2010). However, there is not a definitive understanding of whether pathophysiology of the brain cholinergic system exists in ASD. Related, unknown is whether there are specific brain cholinergic systems that are altered in the disorder and/or if a brain acetylcholine pathophysiology exists and how it may relate to particular symptoms in ASD. Addressing these issues can further our understanding of the etiology of ASD and help develop new effective therapeutics. The employment of animal models can help address these issues. The present findings in the BTBR mouse, a model of idiopathic autism, reveal that the non-specific mAChR agonist, oxotremorine attenuates repetitive motor behaviors without affecting general ambulation. Thus, treatment with a mAChR agonist may be effective in reducing repetitive behaviors in ASD.

## Conflict of interest statement

The authors declare that the research was conducted in the absence of any commercial or financial relationships that could be construed as a potential conflict of interest.
